# A quantitative analysis of gait patterns in vestibular neuritis patients using gyroscope sensor and a continuous walking protocol

**DOI:** 10.1186/1743-0003-11-58

**Published:** 2014-04-11

**Authors:** Soo Chan Kim, Joo Yeon Kim, Hwan Nyeong Lee, Hwan Ho Lee, Jae Hwan Kwon, Nam beom Kim, Mi Joo Kim, Jong Hyun Hwang, Gyu Cheol Han

**Affiliations:** 1 Department of Electrical and Electronic Engineering and Institute for IT Convergence, Hankyong National University, Anseong, South Korea; 2 Department of Otolaryngology-Head and Neck Surgery, Kosin University College of Medicine, Busan, South Korea; 3 Department of Preventive Medicine, Graduate School, Pusan National University, Busan, South Korea; 4 Neuroscience Research Institute, Gachon University, Graduate School of Medicine, Incheon, South Korea; 5 Department of Otorhinolaryngology, Yonsei University College of Medicine, Seoul, South Korea; 6 Department of Otolaryngology-Head and Neck Surgery, Gachon University of Medicine and Science, Graduate School of Medicine, Incheon, South Korea

**Keywords:** Locomotion, Gait, Vestibular system

## Abstract

**Background:**

Locomotion involves an integration of vision, proprioception, and vestibular information. The parieto-insular vestibular cortex is known to affect the supra-spinal rhythm generators, and the vestibular system regulates *anti-*gravity muscle tone of the lower leg in the same side to maintain an upright posture through the extra-pyramidal track. To demonstrate the relationship between locomotion and vestibular function, we evaluated the differences in gait patterns between vestibular neuritis (VN) patients and normal subjects using a gyroscope sensor and long-way walking protocol.

**Methods:**

Gyroscope sensors were attached to both shanks of healthy controls (*n*=10) and age-matched VN patients (*n* = 10). We then asked the participants to walk 88.8 m along a corridor. Through the summation of gait cycle data, we measured gait frequency (Hz), normalized angular velocity (NAV) of each axis for legs, maximum and minimum NAV, up-slope and down-slope of NAV in swing phase, stride-swing-stance time (s), and stance to stride ratio (%).

**Results:**

The most dominant walking frequency in the VN group was not different compared to normal control. The NAVs of *z*-axis (pitch motion) were significantly larger than the others (*x*-, *y*-axis) and the values in VN patients tended to decrease in both legs and the difference of NAV between both group was significant in the *ipsi-*lesion side in the VN group only (*p*=0.03). Additionally, the gait velocity of these individuals was decreased relatively to controls (1.11 ± 0.120 and 0.84 ± 0.061 m/s in control and VN group respectively, *p*<0.01), which seems to be related to the significantly increased stance and stride time of the *ipsi-*lesion side. Moreover, in the VN group, the maximum NAV of the lesion side was less, and the minimum one was higher than control group. Furthermore, the down-slope and up-slope of NAV decreased on the impaired side.

**Conclusion:**

The walking pattern of VN patients was highly phase-dependent, and NAV of pitch motion was significantly decreased in the *ipsi-*lesion side. The change of gait rhythm, stance and stride time, and maximum/minimum NAV of the *ipsi-*lesion side were characteristics of individuals with VN.

## Background

Among the general population, 20-30% of individuals experience unsteadiness in balance that affects daily activities [1]. Disequilibrium and increased magnitude of temporal gait variability correspond to a higher risk of bodily fall, which is becoming a serious social problem for the elderly [2]. Appropriate body motion in space during locomotion depends on the integration of vision, proprioception, and vestibular information [3]. These afferent sensory feedback signals related to locomotion play a crucial role in adapting and modulating the operation of the locomotor network in the real environment [4]. The network of locomotion and stance in the central nervous system is organized hierarchically, i.e., spinal pattern generators are controlled by supra-spinal locomotor centers [5-7]. Recently, a study of functional imaging has shown that locomotion center in brain share the vestibular cortex [8]. Therefore, changes in vestibular function may be one cause of ataxia. For low-speed locomotion, the otolith organ plays a particularly important role as a central pattern generator [9]. As a part of this network, the vestibular organ is directly responsible for adjusting *anti-*gravity muscles in the same side leg through the extrapyramidal track for posture maintenance and proper adjustment of the center of gravity [10,11]. To evaluate the vestibular role in locomotor regulation, it is important to recognize that results of gait analysis may be influenced by internal factors, such as vestibular compensation [12,13], visual function [14], somatosensory capability [15], aging [16-18], and mental alertness [19]. Gait analysis may also be related to external factors, such as the environment in which the tests are performed, medication, nature of the ground surface [20], and number of gait cycles in the walking test [21]. In vestibular-deficient patients, gait analysis exhibits compensatory alterations with respect to step width, gait speed, and duration of double support during walking [22-27]. Vestibular afferent stimulation induces a phase-dependent modulation of leg motor control during locomotion [22]. To prove these findings, traditionally, three basic systems have been used: a motion-capture system, force plates, and electromyography [28]. By use of the three-dimensional position data obtained from motion-capture systems, it is possible to compute joint angle, temporal-spatial, and kinematic data from a walking test of at least 6 m. However, to reliably assess gait variability and evaluate human motor performance, it has been recommended to collect over 10 to hundreds continuous stride in order to avoid the limitations of conventional gait protocols, and to get measuring variability [21,29,30]. To overcome this obstacle, simplified testing devices, such as gyroscope sensors and accelerometers, have been introduced for objective gait analyses [31,32]. Nonetheless, there was not any report the result of gait pattern analysis in patients with vestibular disorder using long way walking protocol yet.

The purpose of the current study is to analyze quantitatively gait patterns in vestibular neuritis patients using a gyroscope sensor and a continuous walking protocol. For the study, vestibular neuritis (VN) patients were analyzed and compared to healthy age- and body- weight-matched individuals.

## Methods

### Subjects

All clinical experiments were carried out from August 2011 to April 2012 with the approval (GIRBA2248) of the Gachon University Institutional Review Board (Incheon, South Korea). We conducted a case–control study with healthy volunteers (y = 44 ± 9.68; n = 10; male/female ratio = 4/6) as the control group and VN patients (y = 45 ± 10.51; n = 10; male/female ratio = 6/4, right-/left-side lesions = 5/5) as the study group. We noted the age (*p* = 0.92), height (VN = 163.3 ± 7.21 cm, control = 161.8 ± 8.59; *p* = 0.99), and weight (VN = 64.3 ± 8.9 kg, control = 59.3 ± 9.6 kg; *p* = 0.240) of all participants. Vestibular neuritis was diagnosed according to the Coates criteria [9]. Recruited patients had been referred to a tertiary hospital (Incheon, South Korea) within 3 days of the onset of their symptoms and had not taken any anti-vertiginous medications. Canal paresis and spontaneous nystagmus values for the VN group were 31.69 ± 12.16% (ranging from 18.89 to 44.03) and 11.46 ± 8.89°/s (ranging from 2.51 to 21.54), respectively. The walking test was administered 7 days after laboratory tests were performed to ensure the patients’ safety. Healthy volunteers recruited from the area of Incheon were defined by absence of any neuro-otologic diseases.

### Walking test protocol

A walking test was performed in a corridor with height, width, and length of 4 m, 10 m, and 88.8 m respectively. The floor was flat and not slippery, and it was constantly illuminated at 100 lux or higher. A 15-cm-wide blue guideline had been drawn on the floor of the corridor along which the subjects were lead while walking. A one-way walk was made at a normal gait speed. Neither left nor right wall of the corridor had any decoration whatsoever, so that visual stimulation of the subjects was minimized. Auditory stimulation was simultaneously minimized since the corridor lacked windows, and efforts were made to reduce any noise during the test. Two test observers accompanied each subject during the test without providing assistance with walking.

### Gyroscope sensing system

A 3-axial ITG3200 gyroscope sensor (InvenSense; Sunnyvale, CA, USA), SDSDB-2048 memory (SanDisk Co., Milpitas, CA, USA), and CR2 rechargeable battery (Panasonic Co., Secaucus, NJ, USA) were compactly integrated into a single device. Three-axial angular velocity data obtained from sensor was sent to the memory unit every 20 ms via an integrated circuit, where the information was stored by a secure digital card; the storage rate was regulated by an Atmega328 microcontroller (Atmel; San Jose, CA, USA). Sensor calibration was performed by comparing the estimated angular velocity with parameters provided by the sensor manufacturer, and velocity values were measured with a commercial inertia sensor (MotionNode, GLI Interactive; Seattle, WA, USA) [33]. The synchronization of each sensor was done by simultaneously shaking the sensors, thus inducing a sudden change in angular velocity. This sudden velocity change was designated as a starting time.

### Sensor placement

**Figure 1:**
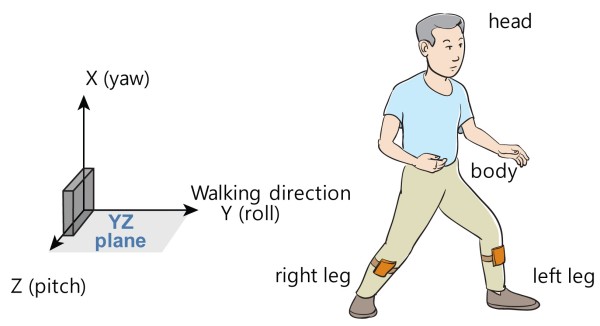
The definition of direction for 3-axis gyro-sensor and sensor position attached to the body.

The sensors were placed on the middle part of both shanks of the study participants. In order to minimize undesired movement of the sensors, Velcro tape (W015) from Shenzhen Dongsanxin Velcro Textiles Co., Ltd. (Guangdong, China) and tensor bandages (JB3301) from Ningbo Jumbo Medical Instruments Co., Ltd. (Zhejiang, China) were used to secure the sensors to the legs. The three axes of all sensors were aligned in the same direction. Walking direction, antigravity direction, and the right side of the subject were defined as the *y-*, *x-*, and *z*-axes, respectively (Figure 1).

### Data processing

The stance phase was defined as the time that the foot was on the ground, and swing phase was the time the foot was in the air. The desired period for analysis was selected using a graphical programming tool (LabVIEW, National Instruments; Austin, TX, USA). Selected time window of the data was analyzed with formulae (1) to quantify relative changes in the three axes. The normalized angular velocity (expressed as a ratio of the angular velocity, NAV) was calculated with the formulae (1) below, after determining the angular velocity difference for each axis relative to “0” angular velocity which represented the stationary state.

Formulae (1):

$$\text{ratio}\;\text{of}\;\text{gx}=\frac{\text{gx}}{\text{gx}+\text{gy}+\text{gz}}$$

$$\text{ratio}\;\text{of}\;\text{gy}=\frac{\text{gy}}{\text{gx}+\text{gy}+\text{gz}}$$

$$\text{ratio}\;\text{of}\;\text{gz}=\frac{\text{gz}}{\text{gx}+\text{gy}+\text{gz}}$$

**Figure 2:**
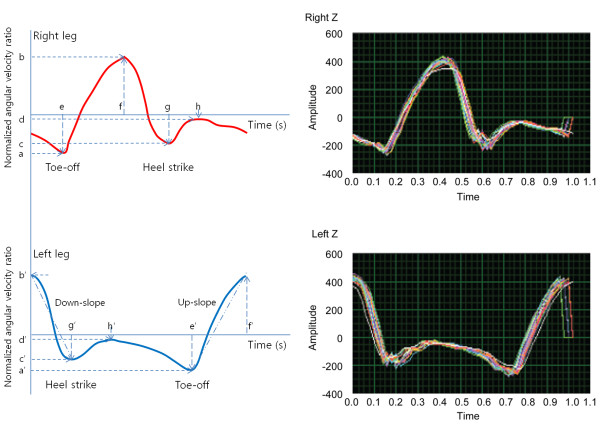
**Parameters definition used for gait analysis.** Right side pictures were from one normal person, and left side diagram present parameters for gait pattern analysis. In the pictures and diagram, the *y*-axis represents the normalized angular velocity (NAV), and the *x*-axis represents time (s). a-a’: NAV for toe-off (=minimum value of NAV); b-b’: NAV of the mid-swing phase (=maximum value of NAV); c-c’: NAV for heel strike; d-d’: NAV for the mid-stance phase; e: time of maximum toe-off; f: time of maximum swing phase; g: time of maximum heel strike; h: time of maximum mid-stance phase. Down-slope means the down-slope of NAV, Up-slope means the up-slope of NAV.

In these expressions, *g*_*x*_ is the angular velocity of the *x*-axis; *g*_*y*_ is the angular velocity of the *y*-axis; and *g*_*z*_ is the angular velocity of the *z*-axis. According to a defined stance and swing phase, cumulative *z*-axis data were plotted on a time-versus-angular velocity graph that was subsequently used as an index for gait analysis in this study (Figure 2).

In the newly defined parameter analysis of Figure 2, a-a’ means the NAV for toe-off (minimum value of NAV); b-b’, the NAV of the mid-swing phase (maximum value of NAV); c-c’, the NAV for heel strike; d-d’, the NAV for mid-stance phase; e, the time of maximum toe-off; f, the time of maximum swing phase; g, the time of maximum heel strike; h, the time of maximum mid-stance phase. To verify the dynamic of NAV over time, parameters as like as the down-slope and up-slope of NAV were defined using the slopes between b’ to g’ and e’ to f’ respectively. The stride time was defined as the value of f’; the stance time, from g’ to e’. Then, the swing time was defined as the subtraction value of the stance time from the stride time.

In human bipedal walking, two legs have reciprocally opposite phases. Therefore, we analyzed the data from perspectives of the left leg or from a lower positioned diagram as in Figure 2.

### Statistical analysis

All statistical analyses were performed using SPSS v17.0 (SPSS Inc.; Chicago, IL, USA). The NAV data were described using a Box-Whisker plot. We also performed Mann–Whitney U tests. The normality of the data was checked with a Kolmogorov-Smirnova test before performing parametric tests. A parametric ANOVA test was used to compare the mean difference of normally distributed gait data, and a Kruskal-Wallis test was used to compare differences of central tendency of the gait data that were not normally distributed. For post-hoc tests, LSD was used for variables having equal variance (maximum and minimum NAV of swing phase, time of stride and swing phase), and Dunnett’s T3 was used for variables having unequal variance (up-slope and down-slope NAV of swing phase). Stance time and ratio were analyzed by a Mann–Whitney U test with Bonferroni correction. We considered differences to be statistically significant when *p* < 0.05.

## Results

### Frequency analysis of gait cycle

**Figure 3:**
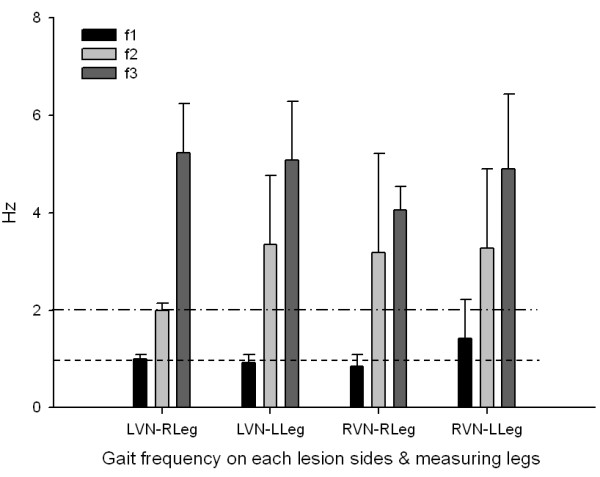
**Analysis of gait frequency on each lesion sides and measuring legs.** Repeated gait cycles were analyzed by frequency domain according to lesion side and measuring leg side. The frequency analysis was done for three main dominant frequencies as f1, f2, and f3 respectively. For the control group, the f1 and f2 were observed at 1.1 ± 0.015 and 2.0 ± 0.014 Hz in the left leg; otherwise, these values were observed at 0.94 ± 0.111 and 3.4 ± 1.627 Hz (*p* < 0.001) in the lesion side leg. RVN and LVN mean vestibular neuritis on each right-and left-side. RLeg and LLeg mean leg side for measuring. Upper and lower broken lines mean the normal value of each f2 and f1.

For the control group, two dominant frequencies of gait cycle were observed at 1.1 ± 0.015 and 2.0 ± 0.014 Hz in f1, f2 respectively on the left leg and these were not significantly different between both legs. In contrast, the first dominant frequencies (f1) of gait cycle in lesion side of VN patients were observed at 0.94 ± 0.111 Hz and the second dominant frequency (f2) was ambiguous and higher in the VN patients (3.4 ± 1.627, *p* < 0.001) than the normal control (Figure 3).

### Axial analysis

**Figure 4:**
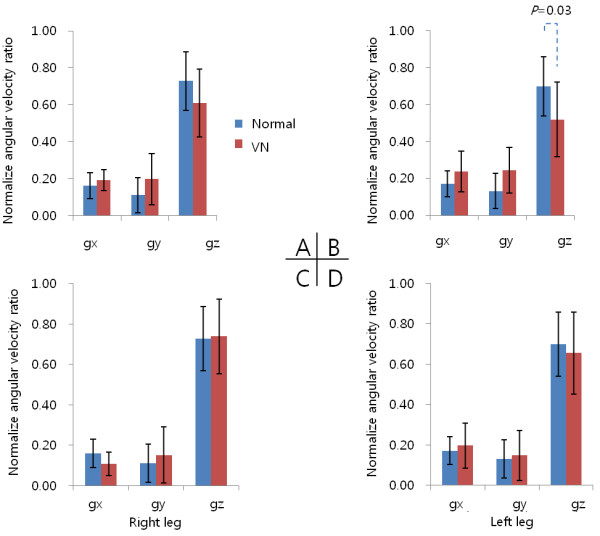
**The normalized angular velocity for each axis of both legs.** Differences between normal individuals and vestibular neuritis (VN) patients are compared according to lesion side. Significant differences in pitch axes for the left leg were observed for left-side VN patients only (*p*=0.03). **A** and **B**: left-side VN. **C** and **D**: right-side VN.

The NAVs of *z*-axis (pitch motion) were significantly larger than the others (*x*-, *y*-axis) (*p* < 0.05). Data for the VN patients were analyzed according to the side on which the lesions occurred. In the VN patients with lesions on the left side, NAV for the *z*-axis (corresponding to the pitch motion) in the *ipsi-*lesion leg was significantly different compared to control group (*p* = 0.03, Mann–Whitney U test). However, this phenomenon was not clear in the group with right-side lesion (Figure 4).

### Gait velocity

Additionally, the gait velocities of these individuals were decreased relatively to controls (1.11 ± 0.120 and 0.84 ± 0.061 m/s in control and VN respectively, *p* < 0.01, Mann–Whitney U test). The numbers of strides were 69.5 ± 2.521 and 99 ± 4.237 in control and VN group respectively.

### Analysis of gait parameters

Each variance (σ^2^) was 0.1116/0.1152 (heel strike, g’ in Figure 2) and 0.273/0.387 (toe-off, e’ in Figure 2) in control/VN group respectively.

Comparison of the left-foot gait between left- and right-side VN patients indicates that the maximum NAV of the swing phase was significantly decreased, and the minimum one was significantly increased, on the impaired side (p < 0.05, LSD). Furthermore, the down-slope and up-slope of NAV were decreased on the impaired side (*p* < 0.05, LSD and Dunnett’s T3 test). In addition, stride and stance time for the impaired side significantly increased (*p* <0.05, LSD and Mann–Whitney U test). Stance ratio was also significantly different according to the side of impairment (*p* < 0.05, Mann–Whitney U test), as summarized in Table 1.

**Table 1:** Summary of gait analysis

**Variable**	**Normal (*n*** **= 10) (mean ± SD)**	**LVN (*n*** **= 5) (mean ± SD)**	**RVN (*n*** **= 5) (mean ± SD)**
Max. NAV^*^	368.6 ± 42.5	258.6 ± 42.3^‡^	349.6 ± 51.1^†‡^
Min. NAV^*^	−172.3 ± 34.5	−125.8 ± 26.9^‡^	−195 ± 47.9^†‡^
Down-slope of NAV^*^	−3003 ± 341.7	−2139.4 ± 401.1^†^	−3251.9 ± 1021.3^†¶^
Up-slope of NAV^*^	2968.7 ± 973.9	1692.6 ± 429^¶^	1893.1 ± 144.5^¶^
Stride time (s)^*^	0.98 ± 0.062	1.15 ± 0.115^†^	1.12 ± 0.069^†^
Swing time (s)^*^	0.389 ± 0.060	0.430 ± 0.053^†^	0.485 ± 0.046
Stance time (s)^**^	0.591 ± 0.044	0.722 ± 0.083^ψ^	0.633 ± 0.059^ψ^
Stance to stride ration (%)^**^	60.4 ± 0.046	62.7 ± 0.030^ψф^	56.6 ± 0.035^ф^

NAV means the normalized angular velocity; RVN, right-side vestibular neuritis (VN) patients; LVN, left-side VN patients. ^*^p < 0.05 (ANOVA). ^**^p < 0.05 (Kruskal-Wallis). ^†^*p* < 0.05 (LSD), comparing normal individuals with RVN or LVN. ^‡^*p* < 0.05 (LSD), comparing LVN and RVN. ^¶^*p* < 0.05 (Dunnett’s T3 test), comparing normal individuals to RVN or LVN. ^ψ^*p* < 0.05 (Mann–Whitney U test), comparing normal individuals to RVN or LVN. ^ф^*p* < 0.05 (Mann–Whitney U test), comparing LVN to RVN.

## Discussion

In the present study using a gyroscope sensor and long way walking protocol, acute changes of vestibular function resulted in an irregular gait rhythm on the *ipsi-*lesion side leg. The VN patients had an elongated stride and stance time on the lesion side, and a slower gait. Additionally, the NAV tended to minimize in these individuals, reflecting that the VN patients were not confident on precise locomotion or experienced fear of possibly falling.

Successful locomotion requires appropriate motor commands for task progression and equilibrium control [34]. Vestibular information likely plays a greater role in tasks in which the relationship between the center of mass and base of support is dynamic [34]. In previous studies, Borel et al. have reported that changes in gait pattern after unilateral vestibular neurotomy (UVN) are characterized by slower gait compared to controls, and the slow gait after UVN is mainly due to step length and step frequency reductions, for both open- and closed- eye conditions, and locomotion speeds. Especially in the acute stage after UVN, locomotor pattern impairments were significantly accentuated [13]. Kubo et al. have reported that stride length and step cycle decreased after caloric stimulation when measured by walking on a treadmill [35]. Finally, a length of step and stride, including gait frequency, is reduced in areflexia and hyporeflexia state of vestibular system and according to walking speed or dual tasking during the test [29,30]. Concerning gait pattern analysis, Lang J et al. reported that vestibular functional impaired patients’ cadence is faster, and the stride time at normal walking speed is shorter than that of the controls, and they have been verified it by 3-D gait analysis [36]. We assumed that the opposite results in previous studies were due to different testing protocol and measurement method. Therefore, we applied the direct measurement of the same gait parameters using the gyroscope sensors on lower legs and long way walking protocol. Our experimental results provide the gait information in the *ipsi-*lesion side of VN patients. In other words, the gait velocity and the stride length were decreased, whereas the stride time and the stride number were increased. Moreover, the gait frequency was not significantly different in the first dominant frequency comparison.

A short way walking protocol or gait analysis using treadmill would be involved in some errors because these methods were not real or not natural [21]. Galna et al. reported that gait variability was more reliable during continuous walks in healthy control and even in people with motion control diseases as like as parkinsonism. Moreover, increasing the number of steps also improved reliability, with the most improvement seen across the first 30 steps [29]. Hollman et al. reported that fewer than 10 to 20 strides are required to reliably measure velocity and cadence in either normal or dual task walking. However, reliable measurements of the stride velocity variability may require hundreds of strides, particularly in the dual task walking [30]. However, generally, conventional devices can only be used to obtain data for subjects walking 6 to 10 m [28]. Thus, only 5 to 8 strides could be assessed, considering that a normal step is about 113.5 cm in stride length [37,38]. Although the main gait frequency was not changed in VN group, moreover, it could not show constant results according to lesion sides. This means the vestibular functional impaired patients’ cadence is unstable and could be modified to prevent falling, according to gait speed and task condition. The wide range of variance (σ^2^) at toe-off timing, and the significant increase of the second dominant frequency support this phenomenon.

With the gyroscope sensors and the long way walking protocol developed in the present study, we were able to acquire sufficient data for investigation of gait velocity and patterns in vestibular loss status.

In other perspective, the vestibular tone can be changed according areflexia to *hypo-* or *hyper-*reflexia. Therefore, the timing of examination reflects the degree of vestibular compensation, and it could be one of the causes of test errors [39]. Eventually, several objective walking tests (a gait Four Square Step Test [40], Timed Up & Go test [41], the circular walking test [42], and an 11-m walk test [40]) and Dynamic Gait Index [43] have same problems to evaluate disability in vestibular disorder. Although we are not able to analyze our results here with respect to exactly reflect the compensation status or evaluation of vestibular disability, as this was not addressed in our experiments, our gait data from VN patients directly measured by our own method may have greater confidence than that obtained using conventional equipment or treadmill. Therefore, our data do provide useful information for understanding vestibular signals itself.

To obtain gyroscope data, a mid-shank level was choose in this study because the pelvis (center of gravity) and both leg measurements also showed significant mean differences between the control and the VN group [pelvis: mean difference = 0.0020, t(16.3) = 2.97, p < 0.009, right leg: mean difference = 0.0025, t(33) = 3.0, p <0.005, left leg: mean difference = 0.0020, t(16.3) = 3.3, p <0.004] and because the strong correlations between measurements taken at head (vestibular signal generator) and pelvis as sensor location were observed for both groups (VN/control, r = 0.68/r = 072) [44]. The data sampling frequency in this study was 50 Hz (every 20 ms). This is enough to explain the gait characteristics because the frequency of leg movements is lower than 2 Hz in case of normal walking. The recommended minimum sampling rate is higher than 30 Hz to see clearly the raw data in time domain [45].

We found that shifts in the center of gravity during gait initiation are significantly different between the VN patients and the healthy individuals. This phenomenon was apparent in our study, through the comparison the variance (σ^2^) at the time of maximum heel strike and the time of maximum toe-off. The timing variance of ‘toe-off’ in VN group was marked higher than control. This experimental result showed that the irregularity of gait initiation is higher in the VN group than normal as in a previous study [24,46].

The usefulness of the pitch axis motion for gait analysis confirmed by electromyography of the soleus muscle [22,34,47,48]. In our study, the pitch motions (g*z*) of both legs showed significant NAV ratio differences compared to rolling (g*x*) or yawing (g*y*). However, for the comparison between the normal and the VN pitch motions, the differences were very slight so that they may not be detected. Especially, in experimental results for *z*-axis, the down-slope and up-slope of NAV in swing phase was significantly decreased. Subsequently, the stance time was also significantly prolonged on the impaired side, resulting in an increased total stride time. Thus, gait speed of the VN patients was generally decreased. All these gait pattern analysis results said that the VN patients were not confident on precise locomotion.

In this experiment, significant differences of NAV between the control and the VN groups were seen in the left-*ipsi-*lesion side only (Figure 4B). This may be related to the finding that a cortical lesion on one side of the brain influences vestibular function on the same side in man, and then is expressed only in the *ipsi*-lesion side through the extrapyramidal tract [49]. Nevertheless, we could not believe this as a very reasonable finding in the right-*ipsi-*lesion (Figure 4C). We assumed that laterality may be one explanation for the presence of functional differences between the lower extremities because all participants preferentially used the right limb during voluntary motor acts in the retrograde survey. Basically, an action toward a goal is carried out by the preferred limb (right limb), while support is provided by the other limb (left limb) [50]. Finally, the right leg move strongly forward to the walking goal and the left leg have to move quickly to support the right leg in the right-limb-preferred person. Therefore, the change of NAV in the right-*ipsi-*lesion was masking with Previc’s neuro-developmental theory [50]. To overcome this obstacle, considering that locomotion depends on the preference, a symmetry index could be used to calculate the NAV in future studies.

There was several study limitations in this study. Here, we focused only on mid-aged individuals. However, the question of age should also be addressed, as a previous study has shown that the brain activation during locomotion and stance is age-dependent. In advanced age, this multisensory activation is the most prominent during standing, less during walking, and the least during running [15]. We only used normal gait speed in this study. The magnitude of gait parameter variability depends on gait speed in a disease-specific manner [51]. Vestibular information is used mainly in slow gait alone [26]. Normal gait speed is variable and depends on individuals. The interpretation of these experimental results should be limited to factors analyzed in our subjects. Walking is recognized as having a periodically repeated pattern of motion that can be analyzed. In particular, this continuously repeated lower limb motion is closely related to the upper body, arms, pelvis, and head motion [34,52,53]. However, our data showed gait analysis results only due to the interaction between different body parts and the lower limbs will be consecutive study issue. In future, to overcome some of these present study limitations, further experiments with increasing number of sensors and use of various walking protocols and electromyography should be conducted because the sensor system and the long way walking test showed valuable results to evaluate the VN patients.

Generally, vestibular disorders are well compensated for several months, and conventional clinical diagnostic tools could not justify the exactly the compensation status [54]. However, body sway and gait disturbance can be felt for a long time after an acute unilateral vestibular neurotomy [13] or in a bilateral vestibular loss [55]. Therefore, our method has a potential to track locomotion capability for compensation status or improvement of vestibular symptoms after rehabilitation exercises [12,55,56]. The vestibulo-ocular reflex (VOR) is primarily used to diagnose vestibular disorders [57]. While behavioral recovery is dramatic, VOR quantitative testing reveals loss and permanent asymmetry of the vestibular system [54,58]. Therefore, the chance of interpretation error could be included, according to the timing of body sway measurement in the double stance only. In addition to the static posture for maintaining balance during locomotion, the walking test may contribute to an ideal treatment of vestibular rehabilitation when the body has a greater chance to lose balance [39,59].

## Conclusion

In the present investigation using a gyroscope sensor and a long way walking protocol, VN patients showed an irregular gait rhythm caused by irregular gait initiation timing. Moreover, gait speed of the VN patients was slower than that of healthy individuals due to a stride elongation and increased stance time on the lesion side. The maximum and minimum NAV of the swing phase were also minimized among the individuals with VN.

## Competing interests

None of the authors have any competing financial interests to declare. The authors alone are responsible for the content and writing of the paper.

## Authors’ contributions

SCK provided the implemented devices. JYK carried out almost human studies, participated in the data analysis and drafted the manuscript. HNL participated in the design of the study. HHL participated in the design of the study. JHK participated in the design of the study. NBK performed the statistical analysis. MJK drawed the figures and helped to draft the manuscript. JHH participated the human studies. All authors read and approved the final manuscript.
